# Beginning on an age‐friendly journey: Barriers to implementing age‐friendly initiatives

**DOI:** 10.1111/ajag.12930

**Published:** 2021-03-16

**Authors:** Stephen Neville, Sara Napier, Kay Shannon, Jeffery Adams

**Affiliations:** ^1^ Department of Nursing Auckland University of Technology Auckland New Zealand; ^2^ School of Clinical Sciences Auckland University of Technology Auckland New Zealand; ^3^ Shore and Whariki Research Centre Massey University Auckland New Zealand

**Keywords:** community, gerontology, healthy aging, policy, social environment

## Abstract

**Objective:**

To explore the barriers to communities in New Zealand developing age‐friendly initiatives.

**Methods:**

A qualitative participatory approach underpinned this study. Semi‐structured digitally recorded individual interviews were undertaken with 24 government officials, local government steering group members and community representatives from an urban city, provincial city and a rural district. A general inductive data analytic process was undertaken. The consolidated criteria for reporting qualitative research (COREQ) guidelines were followed to ensure rigour in this study.

**Results:**

(a) Being at the beginning, (b) Minimal diversity and (c) Problems getting started were three key issues identified.

**Conclusions:**

New Zealand is in the early stages of becoming age‐friendly. Findings from this study provide a place‐based New Zealand perspective and have influenced central government social policy and practice development, culminating in resources supporting local government and communities to successfully implement age‐friendly initiatives.

**Policy Impact**

Empirical data are integral to the development and implementation of policy initiatives. New Zealand is beginning its journey to become age‐friendly. The place‐based findings from this study has supported the development of central government policy initiatives including the development of an age‐friendly advisory board and a set of publicly available resources.

**Practice Impact**

As New Zealand embarks on its quest to be a country for all ages, associated resources need to be made available to local communities that will help guide and support the implementation of age‐friendly initiatives. Requisite support includes practical ‘tips’ that can easily be translated into action.

## 1 INTRODUCTION

The ageing of the world's population is well recognised and reported. Currently, 15% of New Zealanders are aged 65 years and over, and in line with global trends, these statistics are set to continue to increase.^1^ It is estimated that by 2034, 21% of New Zealanders will be aged 65 years and over, with the most significant increases occurring in the 85 and over, as well as indigenous groups.^2^ In addition, the influences of global migration on ageing has seen recent increased numbers of older migrants living in Western countries, including New Zealand.^3^ Therefore, the social and environmental needs of a diverse group of older people need to be considered, visible and integrated into social policy.

Advancing age‐friendly communities has become a priority for governments internationally and in New Zealand in response to the ageing of populations, as well as the development and integration of ageing in place policies. Ageing in place is referred to as supporting older people to continue living in communities of choice, with or without assistance.^4^ Consequently, communities need to be appropriate and supportive environments for older people to live in.

The World Health Organization (WHO) launched the *Global Age‐Friendly Cities: A Guide* in 2007 following a substantial coordinated research project undertaken in 33 cities across 22 countries. Eight themes, identified from consultation with older people and community representatives, were found to be essential for a city to be age‐friendly. These themes relate to outdoor spaces and buildings; transportation; housing; social participation; respect and social inclusion; civic participation and employment; communication and information; and community support and health services.^5^

The age‐friendly cities framework builds on the WHO’s concept of active ageing, which in turn culminated from activities associated with the 1999 United Nations International Year of Older Persons. Active ageing is defined as ‘the process of optimizing opportunities for health, participation and security in order to enhance quality of life as people age’ (p. 12).^6^ The WHO subsequently updated the concept of active ageing to healthy ageing in the 2015 World report on ageing and health.^7^ Following publication of this report, the importance of healthy ageing as a public health priority has been underscored by the WHO along with re‐emphasising the importance of age‐friendly environments.^8^

Internationally, an increasing number of cities and communities have started to implement age‐friendly programs to create supportive environments that promote respect, inclusion, empowerment and participation for older people. Since 2007, 1000 cities and communities throughout 41 countries have joined the age‐friendly Global Network. This global network provides a platform to discuss and share ideas and experiences between cities and communities interested in developing age‐friendly initiatives.^9^

New Zealand has a central government department, the Office for Seniors, responsible to the Minister for Seniors. A key work stream within this department is age‐friendly communities and ensuring neighbourhoods are inclusive for people of all ages. New Zealand is committed to becoming age‐friendly and has joined the WHO Global Network. There are two main government strategies that provide the framework to address the health and well‐being of older New Zealand citizens: The *Healthy Ageing Strategy* ^10^ and the *Better later life: He Oranga Kaumātua 2019‐2036 Strategy*.^2^ Both of these pivotal documents outline the importance of ensuring our communities are age‐friendly and provide policy, education, research and practice direction.

Studies evaluating the implementation of age‐friendly programs have begun to emerge internationally.^11^, ^12^, ^13^, ^14^, ^15^, ^16^ While similar patterns of success factors and barriers to implementing and sustaining age‐friendly programs are found across different settings, recent critique has highlighted the importance of building place‐based process evaluations to inform the integral continuous improvement process. Further, studies of various age‐friendly approaches from diverse social, political and cultural contexts will enable comparative studies.^17^, ^18^

In summary, New Zealand is in the early stages of working towards being age‐friendly and as such is supporting communities and researchers to provide a sound evidence base for future policy and guideline development. Drawing on New Zealand data, this article presents research findings exploring the barriers to communities developing age‐friendly initiatives. In doing so, these findings contribute to local and central government policies that support the ongoing development of other communities in their quest to become age‐friendly. In addition, findings are available to contribute to the WHO policy agenda through reporting empirically based evidence. Hence, the objective of this study is to explore the barriers to communities in New Zealand developing age‐friendly initiatives.

## 2 METHODS

From mid‐2015, the Minister for Seniors, via the Office for Seniors, began working in partnership with‐ and funded ‐ three distinct pilot communities, to support and promote the development, as well as implementation, of age‐friendly initiatives. The three pilot sites included an urban city (referred to as ‘H’), a small provincial city (referred to as NP) and a rural district comprised of a number of small towns (referred to as K). It was hypothesised that these three sites would undertake a variety of approaches to the implementation of the age‐friendly initiatives, which then could be useful to inform the activities of other communities when implementing their own age‐friendly initiatives. Drawing on findings from these three communities, the aim of this study was to identify the barriers that impede the development of age‐friendly initiatives. When developing this manuscript, we utilised the Consolidated criteria for reporting qualitative studies (COREQ), a 32‐item checklist to report the study.^19^

### 2.1 Study design

A qualitative participatory research approach provided the framework for addressing the research aim. The central tenets of participatory research methodologies are to ensure the active engagement of participants in all aspects of the research process. This requires the development of meaningful partnerships between researchers and participants.^20^ Outcomes of participatory studies should culminate in action.

An interview protocol was jointly developed by the research team and key stakeholders. Due to geographical spread, this participatory process occurred using a secure online platform. The WHO^6^ age‐friendly principles guided the development of key topic areas to be explored during the interviews. These related to the social, structural and political barriers to initiating age‐friendly programs/activities.

### 2.2 Participants and recruitment

In accordance with participatory research principles, key stakeholders were involved in determining the focus and then development of the research project. This included suggestions on the groups of people most appropriate to be approached to participate in the study. A purposeful and targeted recruitment strategy was employed. This approach ensured participants were knowledgeable about the issues related to implementing age‐friendly strategies within their communities. A cover letter was sent to potential informants inviting them to participate, and those interested contacted the lead researcher via email. Inclusion criteria for this study were those employed in central or local government roles related to supporting the development of age‐friendly communities, as well as older people who were members of age‐friendly committees in each of the three study settings. An electronic information sheet was sent and a time negotiated to undertake a qualitative semi‐structured interview.

### 2.3 Data collection

Due to the geographical distances between study sites, interviews were undertaken in a variety of ways such as in person at a place choice, as well as via telephone or Skype. Prior to the interview taking place, any questions related to the research project were answered and consent given. Written consent was obtained from those undertaking face‐to‐face interviews and email consent received from participants being interviewed via telephone or Skype.

Three members of the research team undertook the participant interviews. Each interviewer was provided with an interview guide to ensure the key topic areas were addressed. Examples of questions asked included ‘To what extent have Māori been engaged with when developing age‐friendly programs in your community’, ‘Tell me about the quality of support your community has received from central and local government’ and ‘How aware do you think your community is of the WHO age‐friendly concepts’?

All interviews were digitally recorded and transcribed verbatim by a transcriber who signed a confidentiality agreement. Interviews were undertaken in a place of choice determined by the participants and were approximately one hour in duration, continuing until data saturation was reached. This project was reviewed and approved by Auckland University of Technology Ethics Committee (AUTEC 17/404) on 1 December 2017.

### 2.4 Data analysis

Following transcription, data were analysed using a general inductive approach.^21^ This was an appropriate method that enabled the establishment of clear links between the research focus and data. All members of the research team participated in the data analytic process. Firstly, the transcripts were read individually by all members of the research team and key relevant categories of interest were identified. The research team then presented, and discussed provisional categories. Final categories were then determined and agreed on by all researchers. These main findings were presented and discussed with key stakeholders at an online sense‐making session and were used to inform the recommendations for the future development of age‐friendly communities.

## 3 RESULTS

A total of 22 people agreed and participated in the interviews. These were comprised of four central and three local government officials, six older people who were members of local government age‐friendly steering groups and nine older community representatives. Participants were equally representative of the three pilot communities supported by the Office for Seniors.

Barriers to developing age‐friendly initiatives were evident across each of the study sites and are represented in the following categories; ‘Being at the beginning’, ‘Minimal diversity’ and ‘Problems getting started’.

### 3.1 Being at the beginning

Two aspects that reflect New Zealand being at the beginning of the age‐friendly process were identified by participants. These relate to providing appropriate support to communities and developing a consistent understanding of what constitutes age‐friendliness. Commitment and engagement by central and local governments, as well as communities, was seen as integral to the successful development of age‐friendly initiatives. At a central government level, it was clear that, although supportive, they were not adequately resourced and therefore did not have the capacity to support communities in their quest to become age‐friendly.

[Central Government] are really supportive, but they are under‐resourced. Ageing is a significant issue for our country, but the Office, which is part of a larger Ministry, is underfunded. They just don’t have enough resources to support our communities. (H steering group member)

Due to a lack of funding at central government level, participants identified there was limited ability to provide the necessary resources and support for local government and communities to support age‐friendly initiatives.

Some other countries have robust toolkits and frameworks which includes professional support for local councils, running training workshops for communities wanting to be age‐friendly. Currently, there is a website with some available resources but these need to be more extensive and are not enough to help us. (NP local government member)

There was unanimous agreement across each of the three communities that local governments did not always understand what constitutes age‐friendliness. Consequently, strategic planning activities did not include or prioritise ensuring their communities were appropriate places for older people to age in.

There is a lack of understanding around what age‐friendly is within council. Councils need to accept that our population is ageing and get on board with making age‐friendly a priority. Age‐friendly needs to be included in future council plans. At this stage, I think we are really on the back foot. A fear of mine is that we will have an unusable community for older people. (NP steering group member)

Lack of knowledge can lead to negative attitudes towards older people and misunderstandings about what being age‐friendly is. Both factors can contribute to lack of support for the adoption of age‐friendly initiatives.

European society tends to treat older people as useless and stupid. I found some quite disrespectful attitudes from some members of our council and from within the local community. They really just feel that this is older people whining, being self‐entitled and they [older people] had it all and all they did was ruin the planet. I don’t think it’s recognised just how vulnerable older people are. (K community member)

### 3.2 Minimal diversity

The difficulty in capturing a wide range of perspectives from all members living in the three communities was recognised as a barrier to implementing age‐friendly initiatives. Appropriately engaging with diverse community groups has been identified as essential in forming an inclusive age‐friendly plan.^22^ Undertaking a needs assessment was utilised by each of the communities to capture the views of older people. Overall, participants identified that it was the New Zealand European/Pākehā (the dominant ethnic group), politically motivated and well‐connected community members that had largely influenced the content of age‐friendly plans.

I think only a section of our community was engaged with, mainly those who were white [of European descent], articulate and connected. There were many others who for one reason or another were not consulted, for example, Māori and those from other ethnic and cultural groups [cultural groups included those who were marginalised]. (K community member)

New Zealand is a bicultural country, the partners being Māori (the indigenous people of New Zealand) and New Zealand European/Pākehā. Across each of the three study sites, there was a perception that Māori had not been adequately consulted and involved in the process of developing age‐friendly initiatives.

In terms of our Māori engagement … because generally when we have a project like this, we would approach [name of Māori iwi] and have discussions with them. But they are going through a bit of a restructure at the moment, so that wasn’t an easy process for us. In other words, we didn’t do it. (H steering group member)

This was supported in another community where it was identified that Māori and migrant groups were not involved in the consultation process and their views were not represented in their community age‐friendly plans. This was despite large numbers of people identifying as Māori or being from migrant communities living in these communities.

When the age‐friendly steering group decided to initiate some work, there wasn’t a clear process for engaging Māori or migrant groups, so that was a major disadvantage for what we did. Overall, I would have to say that neither of these groups have been adequately consulted and we don’t necessarily know what they want or need to be age‐friendly. Definitely more work needs to be undertaken. (K steering group member)

### 3.3 Problems getting started

Once a community decided to become age‐friendly, a key challenge was how to begin the process. Each of the local councils in the three study sites already had existing older person steering groups before embarking on the age‐friendly journey. There was unanimous agreement across participants that individual personalities and challenging group dynamics were barriers to getting started.

It took a long time for us to get going, we were paralysed with our initial meetings being glorified talk fests resulting in virtually no concrete outcomes. I think this was largely due to a number of strong personalities in the older population who had very fixed ideas. Therefore, coming to an agreement was a major hurdle. (K community member)

A key issue amongst the steering group and community members was the amount of new and foreign information they were confronted with having to assimilate. The scope of the age‐friendly model was reported as being broad and daunting. Consequently, both of these issues negatively impacted on progress with age‐friendly planning.

People got bogged down in the detail and went around in circles. When you look at the age‐friendly model, it’s huge and it can be really daunting and can derail people which impacts on being able to make progress. We struggled to get started and really needed some clear support and direction from local and central government. (H community member)

Problems getting started were also exacerbated by the heavy reliance on volunteers. Keeping volunteers motivated and involved over the time to progress age‐friendly initiatives was challenging and evident across each of the study sites.

We find it extremely difficult to get a diverse group of community people to put their hand up and participate. Even when people volunteer, it is hard to get people to take the lead on particular projects. It just seems they are happy to make comments but don’t seem able or willing to follow through and do something. (K steering group member)

A frequently occurring concern was the significant amount of time required of an older person volunteering to participate as a steering group member. As identified in the following excerpt, the time commitment required is over a long period of time.

I probably spent at least one day a week for two years doing this, and that’s a lot of time. We have to be aware that some older people may not want to be burdened with committing vast amounts of time and energy over a long period of time. (H steering group member)

## 4 DISCUSSION

New Zealand has only recently committed to becoming age‐friendly. Consequently, there is minimal empirical evidence to support ongoing policy development at central and local government levels. International policy already dictates that a bottom‐up and top‐down approach to ensure the success of age‐friendly initiatives is imperative.^23^ This requires communities, local and central government to work together. The present research interviewed a cross‐section of representatives from central and local governments, as well as community members, identifying key barriers that will inform future social policy development.

Historically, there has been a lack of community voice, particularly the views of older adults, in influencing neighbourhood and community initiatives; yet older people often have lived in their communities for significant periods of time and understand what facilities and opportunities are needed to be age‐friendly. Consequently, the success of age‐friendly initiatives requires the active participation and inclusion of older adult's views and committed leadership^14^, ^16^ to keep the momentum going.^24^ In New Zealand, local council elections have caused disruption to progressing age‐friendly initiatives when changes to the mayor and councillors resulted in the knowledge about, and commitment to the age‐friendly model not remaining consistent over time. This challenge is also mirrored in the Australian and Canadian context.^11^, ^14^

Engagement with Māori and migrant groups was deficient in each of the study sites indicating an urgent need to develop clear processes for appropriately engaging Maori in age‐friendly initiatives. Doing so honours the commitment to genuine bicultural engagement as identified in Te Tiriti o Waitangi (founding legislative document of New Zealand) and legislated at central government level. Meaningful engagement with indigenous groups is particularly salient due to significant changes to New Zealand demography including a predicted ageing Māori population.^25^ In addition, as previously mentioned, there are considerable increases in numbers of older migrant peoples. Direct references to ensuring strong and appropriate Māori and migrant representation on local government age‐friendly steering groups needs to be clearly stated in age‐friendly policy documents. Recent age‐friendly literature has emphasised the imperative to address the diverse needs of older people.^26^ The current findings contribute the early experiences from a uniquely New Zealand bicultural context.

In the present study, resourcing was identified as a barrier to developing age‐friendly initiatives, a finding that resonates with other studies.^16^, ^27^, ^28^ These included a lack of financial backing that impeded the ability to provide resources to guide communities through the processes of developing and operationalising age‐friendly initiatives. Appropriate resourcing is likely to decrease the time burden on volunteers, for example employing a paid project manager and support staff, all of whom should be older.

Findings from this study have influenced central government policy supporting the development of age‐friendly communities. Firstly, the establishment of a central government ‘Age‐friendly Advisory Board’ whose central function is to promote an understanding about age‐friendly communities and to determine a New Zealand‐specific culturally responsive framework and branding that aligns with the WHO model. Secondly, the development of a publicly available age‐friendly portal on the central government, Office for Seniors website. This portal provides information including New Zealand‐specific age‐friendly branding and useful ‘tips’ to guide local government and communities as they begin their journey to becoming age‐friendly.^29^

New Zealand is just beginning the journey to becoming age‐friendly. The importance of this research is to provide central government with empirical data that guides social policy and practice development to better support local government and communities to successfully implement age‐friendly initiatives. A set of resources have already been developed and made publicly available, as well as the formation of an advisory board. These initiatives demonstrate tangible social policy outcomes that have a direct and positive impact on the well‐being of older adults.

### 4.1 Limitations

As with any study there are limitations. While these findings are contextualised to the New Zealand setting, age‐friendliness is a global phenomenon. Consequently, these findings may be transferable and of use to other countries. The three communities involved in this study were all engaged and committed to implementing age‐friendly initiatives. Future studies should include a more diverse range of communities to ensure better representation and therefore understanding of the barriers to implementing age‐friendly initiatives. Finally, data were collected at one point in time and these findings could be further strengthened by undertaking a longitudinal study.

## 5 CONCLUSIONS

This article has presented the barriers identified by key informants about the implementation of age‐friendly communities in New Zealand. The findings demonstrate the importance of including a diverse range of community voices including the authentic engagement of indigenous and migrant peoples. This research has informed the development of tangible central government policy and practice initiatives and resources. These outcomes have a direct and positive impact on the well‐being of older adults.

## CONFLICTS OF INTEREST

No conflicts of interest declared.
